# Analytical techniques for mapping multi-hazard with geo-environmental modeling approaches and UAV images

**DOI:** 10.1038/s41598-022-18757-w

**Published:** 2022-09-02

**Authors:** Narges Kariminejad, Hamid Reza Pourghasemi, Mohsen Hosseinalizadeh

**Affiliations:** 1 Department of Natural Resources and Environmental Engineering, College of Agriculture, Shiraz University, Shiraz, 71441-13131 Iran; 2 Department of Arid Zone Management, Gorgan University of Agricultural Sciences and Natural Resources, Gorgān, Iran

**Keywords:** Environmental sciences, Natural hazards

## Abstract

The quantitative spatial analysis is a strong tool for the study of natural hazards and their interactions. Over the last decades, a range of techniques have been exceedingly used in spatial analysis, especially applying GIS and R software. In the present paper, the multi-hazard susceptibility maps compared in 2020 and 2021 using an array of data mining techniques, GIS tools, and Unmanned aerial vehicles. The produced maps imply the most effective morphometric parameters on collapsed pipes, gully heads, and landslides using the linear regression model. The multi-hazard maps prepared using seven classifiers of Boosted regression tree (BRT), Flexible discriminant analysis (FDA), Multivariate adaptive regression spline (MARS), Mixture discriminant analysis (MDA), Random forest (RF), Generalized linear model (GLM), and Support vector machine (SVM). The results of each model revealed that the greatest percentage of the study region was low susceptible to collapsed pipes, landslides, and gully heads, respectively. The results of the multi-hazard models represented that 52.22% and 48.18% of the study region were not susceptible to any hazards in 2020 and 2021, while 6.19% (2020) and 7.39% (2021) of the region were at the risk of all compound events. The validation results indicate the area under the receiver operating characteristic curve of all applied models was more than 0.70 for the landform susceptibility maps in 2020 and 2021. It was found where multiple events co-exist, what their potential interrelated effects are or how they interact jointly. It is the direction to take in the future to determine the combined effect of multi-hazards so that policymakers can have a better attitude toward sustainable management of environmental landscapes and support socio-economic development.

## Introduction

One natural event may trigger or increase the probability of the occurrence of one or more other natural hazards^1–3^. For example, VanDine and Bovis^4^ and Lucà et al.^5^ suggested that landslides occur in gullies due to the volumetric sediment concentration. The authors also explained that debris flows could influence gully heads on a steeper slope. Meanwhile, Kukemilks and Saks^6^ reported that landslides can occasionally form and develop on the gully bed, improving close relationships. Indeed, Landslides are defined by the down-slope movement of debris, rock, and soil mainly caused by land gravity. They are classified according to their movement type and composite materials^7,8^. Piping erosion divided into two groups, including closed depressions and sinkholes, is also defined as one hazard that has a complex interaction with gully heads^9^ and landslides. Closed depressions initiated when the soil surface steadily lowered above a surface with no break in the vegetation cover^10^; these can eventually change into sinkholes. Sinkholes developed when the surface soil material was plainly interrupted and collapsed^11^. Gully heads are also defined as a natural, mostly vertical fall of the gully channel wall^12^. They are often treated as independent or isolated. Further, there is a need for an alternative (a multi-hazard approach) to recognize all feasible natural events and their interrelationships.

The eventuality of happening collapsed pipes, gully heads, and landslides poses a substantial environmental and physical threat to the general public of arid and semi-arid regions^13^. Their measuring, modeling, and monitoring are not consistently economically or technically feasible; thus, quantitative susceptibility assessment considering “multi-hazard scenarios” and their probable outcomes is becoming a research question and developing a framework for future studies^14,15^. A multi-hazard chain is a series of events that happen in a successional trend triggered by one natural event and temporary and spatial results in the expansion of desertification and land degradation^16–19^. There may also be an interaction between these component natural hazards and even anthropic processes. Further, the term multi-hazards as one of the leading global politics within the aims of disaster-management^20–23^, should be considered by complete multi-hazard research to gain environmental susceptibility in hazardous-prone regions^22^. To mitigate economic losses and decrease the loss of human being life, hotspot zones of multi-hazards should be managed and evaluated. The importance of integrated multi-hazard assessment disaster management, including prevention, preparation, and response was stated by United Nations in 2002. The studies on multi-hazard events have enhanced over the last decades^1–3^. While most previous research has concentrated on individual natural hazards, their relationship to each other has been ignored^19^.

The vast majority of reports regarding the application of data mining in collapsed pipes, gully heads, and landslides studies focused on static modeling and mapping^24,25^, such as “linear regression”^26^, “multiple adaptive regression splines”^27,28^, and “generalized additive models”^29,30^, which several scientists have used. Some other predicting models are also applied for various event susceptibility evaluations with lower restrictions, such as “support vector machines”^31,32^, “decision trees”^27^, “boosted regression tree”^33,34^, “random forest”^9,35–37^, “multivariate adaptive regression spline”^38,39^, and “generalized linear model”^40–42^. Regardless of the method, the recent improvement of satellite-based monitoring tools, “unmanned aerial vehicle UAV”, and advances in numerical spatial modeling^19^, these integrated concepts have provided an impressive tool for multi-hazards prediction and mitigating their impacts^19^. This accurate geometry-based technique overcomes the science–practice gap of lack of detailed analyses of datasets in the susceptibility mapping of soil landforms, and thus, provides noteworthy new knowledge for conservation strategies and targeted management actions in a region which is consistent with a global soil change in environmental exposure^43^. By applying ultrahigh-resolution UAV imagery in the present study, seven types of data mining techniques, i.e., “BRT, FDA, MARS, MDA, RF, GLM, and SVM” were studied for spatial mapping of collapsed pipes, gully heads, and landslides in 2020 and 2021. The multi-hazards maps were prepared and compared for these three hazards in two running years with torrential rainfall. Then, the evaluation for the multi-hazard models was also provided using the receiver operating characteristic curve criterion.

## Results

### “Multicollinearity” analysis using the linear regression algorithm

The linear regression's primary purpose was to recognize the multicollinearity among the corresponding factors. The resulting multicollinearity analysis for three natural events of gully heads, collapsed pipes, and landslides in 2021 are presented in Tables 1, 2 and 3. The results showed that the “TOL and VIF” of all corresponding factors were less than 0.1 and more than 10, respectively. For collapsed pipe events, land use (0.33), silt content (0.21), and slope degree (0.07) had the highest values of beta which represented the higher slope of the line between the important variables and the collapsed pipes (Table 1). The highest beta values related to the occurrence of gully heads were allocated to slope degree (0.33), land use (0.20), and also drainage density 0.03) (Table 2). It showed that the change of these standard deviations in the environmental variables results in standard deviations enhanced in the gully heads. Moreover, multilinearity analysis for the landslide occurrence assigned the greatest beta values to slope degree (0.69), followed by drainage density (0.07), and TWI (0.08), (Table 3).

**Table 1:** Multicollinearity analysis for Collapsed pipes in 2021.

Coefficients^a^							
Model	Unstandardized coefficients		Standardized coefficients	t	Sig.	Collinearity statistics	
	B	Std. error	Beta			Tolerance	VIF
(Constant)	7.973	2.467		3.232	0.001		
Silt content	0.043	0.009	0.210	4.799	0.000	0.814	1.229
OC	− 0.595	0.320	− 0.104	− 1.858	0.064	0.499	2.005
Land use	0.388	0.054	0.333	7.244	0.000	0.736	1.358
ESP	− 0.839	0.268	− 0.185	− 3.132	0.002	0.446	2.243
Distance to streams	− 0.023	0.002	− 0.430	− 10.301	0.000	0.890	1.124
DEM/altitude	0.000	0.000	− 0.098	− 1.865	0.063	0.557	1.795
Drainage density	− 0.019	0.015	− 0.055	− 1.269	0.205	0.828	1.208
CCE	− 0.007	0.021	− 0.015	− 0.325	0.745	0.725	1.379
Soil stability	− 0.301	0.304	− 0.053	− 0.990	0.323	0.543	1.842
Bulk density	− 2.541	1.368	− 0.091	− 1.857	0.064	0.643	1.554
Slope degree	0.142	0.085	0.074	1.668	0.096	0.787	1.271

CCE, calcium carbonate equivalent; OC, organic carbon; ESP, exchangeable sodium percentage.

^a^Dependent variable: collapsed pipes 2021.

**Table 2:** Multicollinearity analysis for gully heads in 2021.

Coefficients^a^							
Model	Unstandardized coefficients		Standardized coefficients	t	Sig.	Collinearity statistics	
	B	Std. error	Beta			Tolerance	VIF
(Constant)	8.266	5.093		1.623	0.107		
Soil stability	− 0.573	0.787	− 0.087	− 0.729	0.467	0.309	3.233
Slope degree	0.661	0.153	0.329	4.306	0.000	0.747	1.338
Silt content	− 0.045	0.020	− 0.174	− 2.267	0.025	0.741	1.350
OC	− 0.858	0.561	− 0.125	− 1.529	0.129	0.652	1.535
Land use	0.237	0.089	0.197	2.667	0.009	0.805	1.242
ESP	− 0.381	0.330	− 0.105	− 1.155	0.250	0.528	1.895
DEM/altitude	0.000	0.001	− 0.067	− 0.719	0.473	0.504	1.983
CCE	− 0.022	0.033	− 0.053	− 0.660	0.511	0.687	1.456
Distance to streams	− 0.020	0.004	− 0.361	− 5.164	0.000	0.896	1.116
Bulk density	− 2.566	3.126	− 0.085	− 0.821	0.413	0.412	2.427
Drainage density	0.010	0.026	0.028	0.400	0.690	0.885	1.130

CCE,calcium carbonate equivalent; OC, organic carbon; ESP, exchangeable sodium percentage.

^a^Dependent variable: Gully heads 2021.

**Table 3:** Multicollinearity analysis for Landslides in 2021.

Coefficients^a^							
Model	Unstandardized coefficients		Standardized coefficients	t	Sig.	Collinearity statistics	
	B	Std. Error	Beta			Tolerance	VIF
(Constant)	− 0.042	0.347		− 0.122	0.903		
Distance to stream	0.001	0.003	0.013	0.229	0.819	0.758	1.320
Drainage density	0.030	0.024	0.069	1.207	0.229	0.753	1.328
Slope degree	1.276	0.149	0.687	8.576	0.000	0.379	2.637
Road distance	0.000	0.000	0.095	1.718	0.087	0.791	1.264
Profile curveture	0.000	0.005	− 0.011	− 0.169	0.866	0.565	1.769
Plan curveture	− 0.008	0.005	− 0.112	− 1.706	0.089	0.566	1.766
Land use	− 0.044	0.087	− 0.030	− 0.510	0.610	0.714	1.400
DEM/altitude	− 0.001	0.000	− 0.137	− 2.431	0.016	0.773	1.294
TWI	0.021	0.019	0.084	1.086	0.279	0.410	2.440

TWI, topographic wetness index.

^a^Dependent variable: Landslides 2021.

### Providing multi-hazard maps in 2020 and 2021

**Figure 1:**
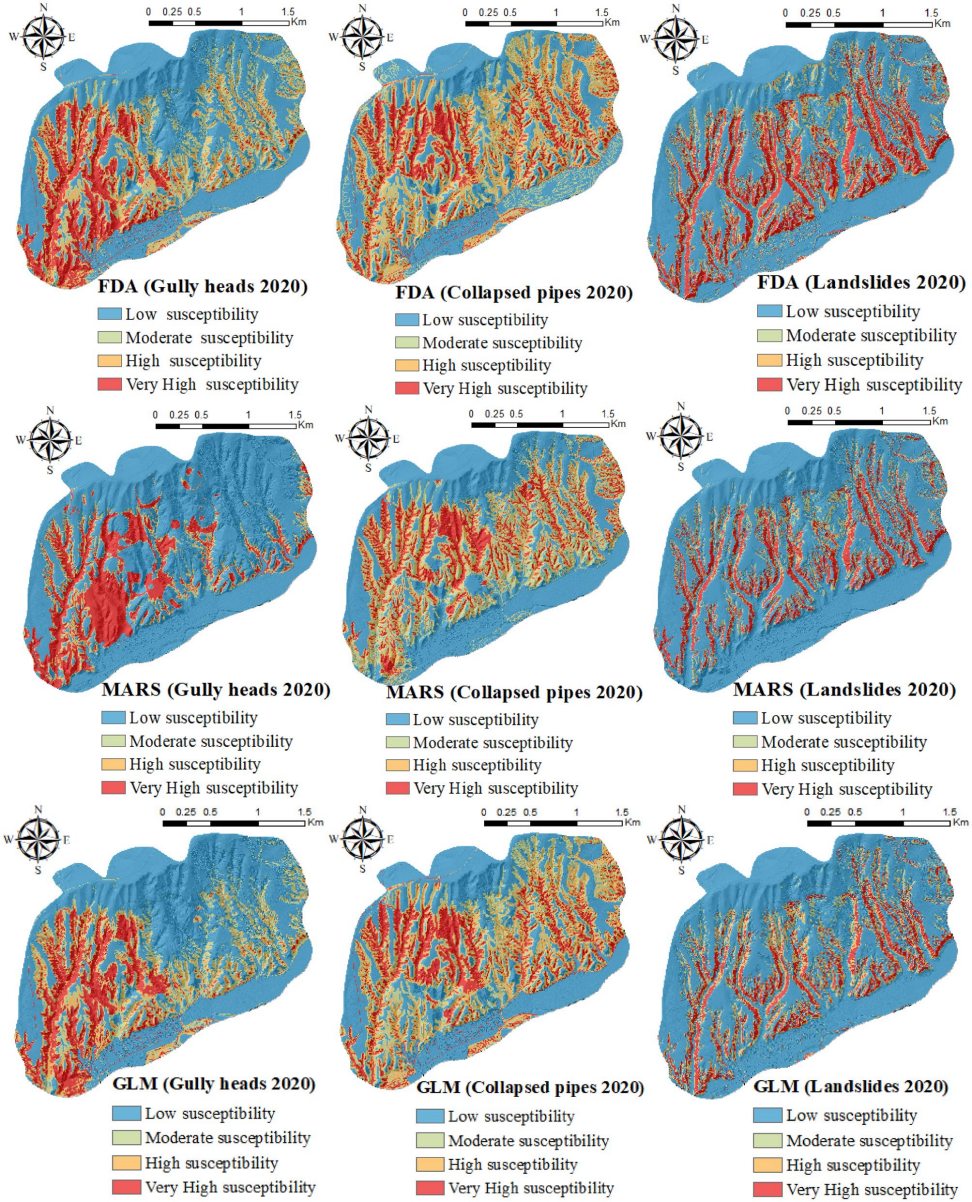
The susceptibility maps of three natural hazards produced using ArcGIS 10.3.1 software (https://www.esri.com) in 2020.

**Figure 2:**
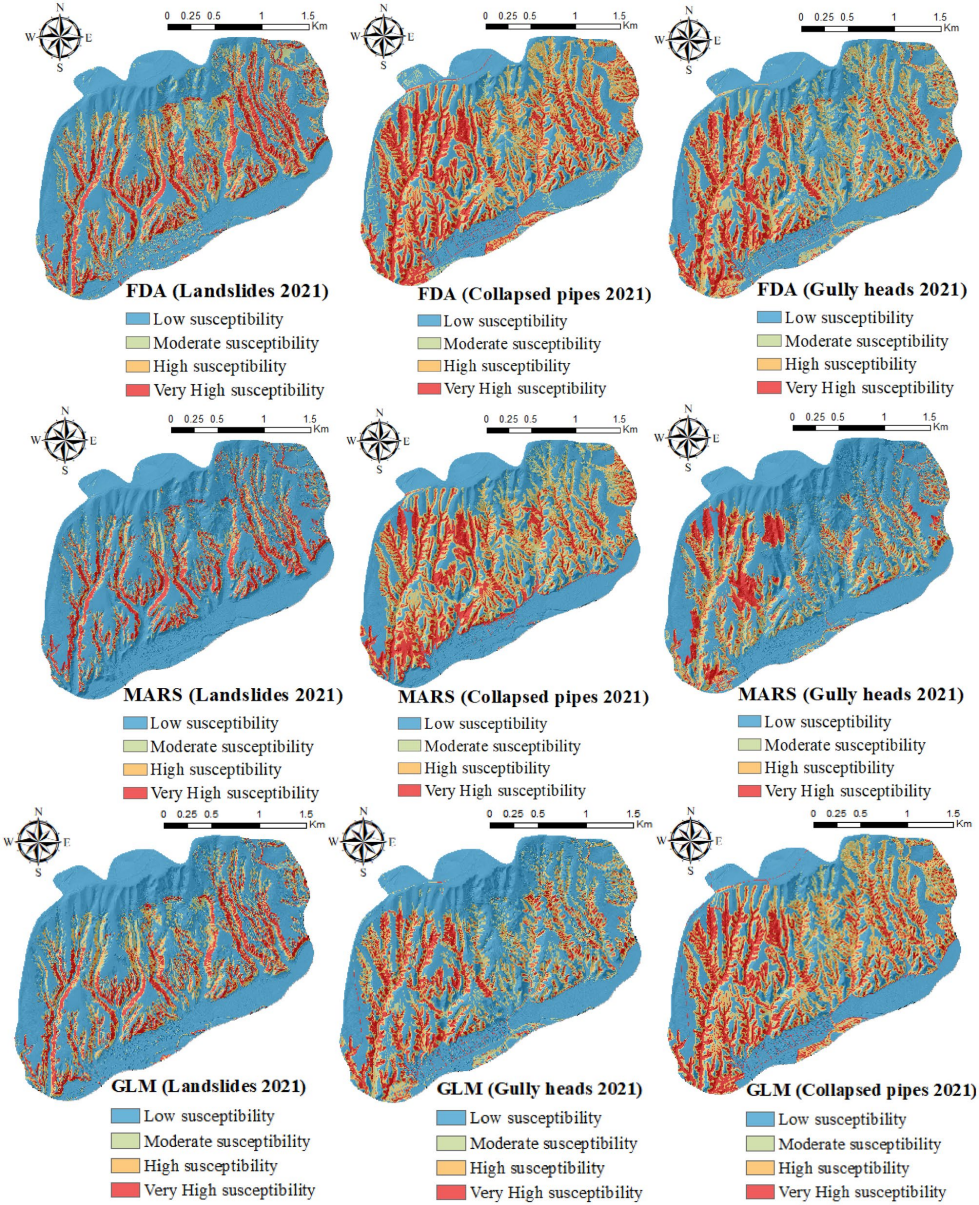
The susceptibility maps of three natural hazards produced using ArcGIS 10.3.1 software (https://www.esri.com) in 2021.

**Figure 3:**
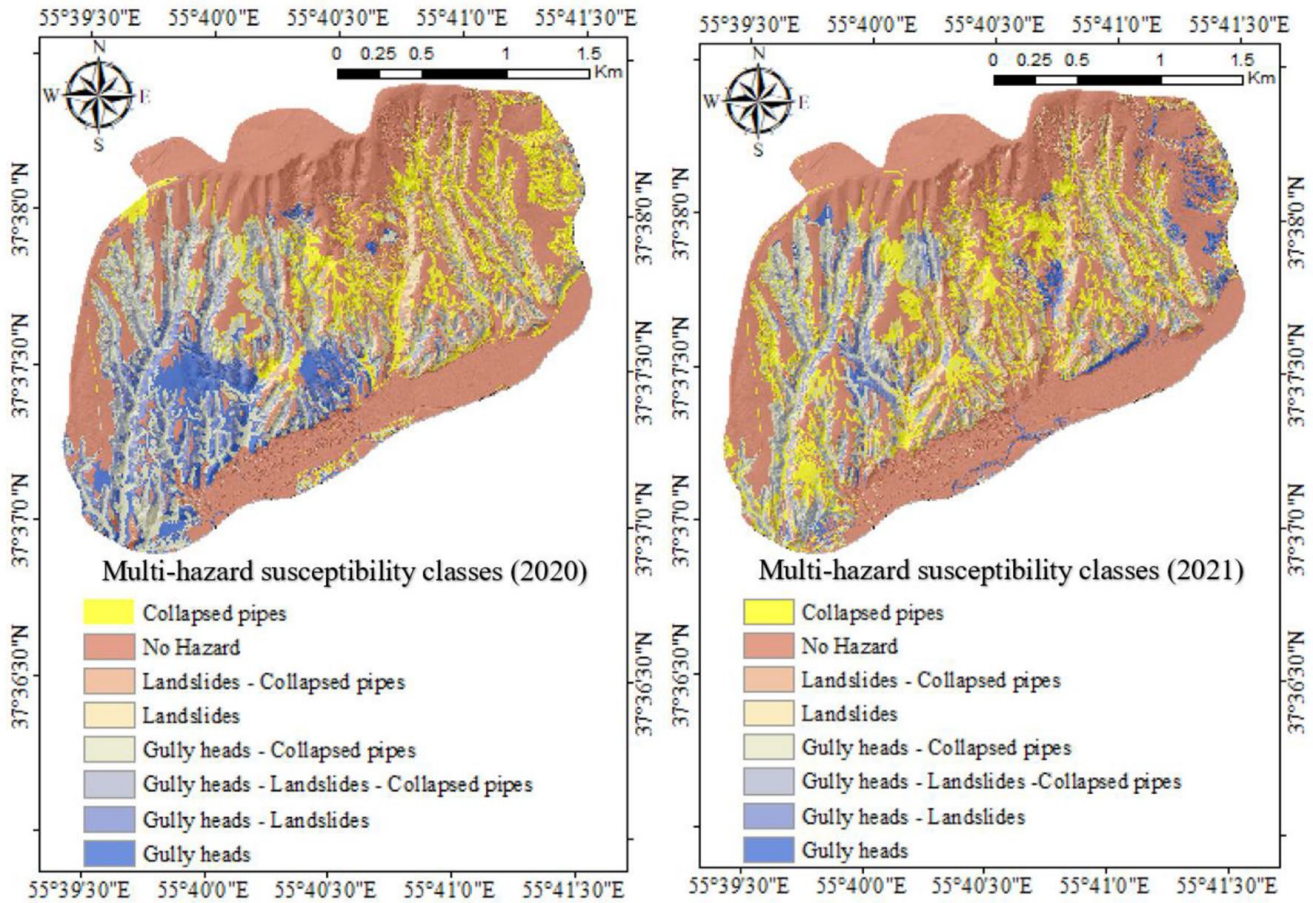
The susceptibility classes of multi-hazard prepared using ArcGIS 10.3.1 software (https://www.esri.com ) in 2020 and 2021.

This section described the susceptibility maps created for three studied hazards using MARS, BRT, FDA, MDA, GLM, RF, and SVM. Figures 2 and 3 showed the susceptibility maps of three natural hazards (i.e., gully heads, collapsed pipes, and landslides) produced using the FDA, MARS, and GLM models in 2020 and 2021. As it is shown in these two figures, the percentage of moderate and high susceptibility classes for all models increased in 2021 compared to 2020. In contrast, the percentage of low and very high susceptibility classes decreased from 2020 to 2021. For example, according to the gully heads susceptibility maps analyzed by MARS in 2020, 70.52%, 6.12%, 5.51%, and 17.85% of the total area were classified into “low, moderate, high, and very high classes”, respectively (Fig. 1). Although, susceptibility classes of gully heads created by the MARS classifier in 2021 were 65.76% (low), 13.30% (moderate), 9.61% (high), and 11.33% (very high) (Fig. 2). Regarding the landslide susceptibility map using GLM in 2020, the greatest percentage of the region (69.92%) had low susceptibility compared with moderate (8.86%), high (8.61%), and very high (12.54%) (Fig. 2). In 2021, the percentage of low (67.21%) and very high (11.98%) landslide susceptibility classes decreased, although an increase was observed in moderate (10.63%) and high (10.11%) susceptibility classes. Based on the collapsed pipes susceptibility map results in 2020, 36.12%, 20.23%, 22.74%, and 20.90% of the region were classified into “low, moderate, high, and very high classes”, respectively (Fig. 1). In 2021, low (38.27%) and very high (22.80%) classes were enhanced, while moderate (18.44%) and high (20.49%) classes were reduced (Fig. 2).

**Figure 4:**
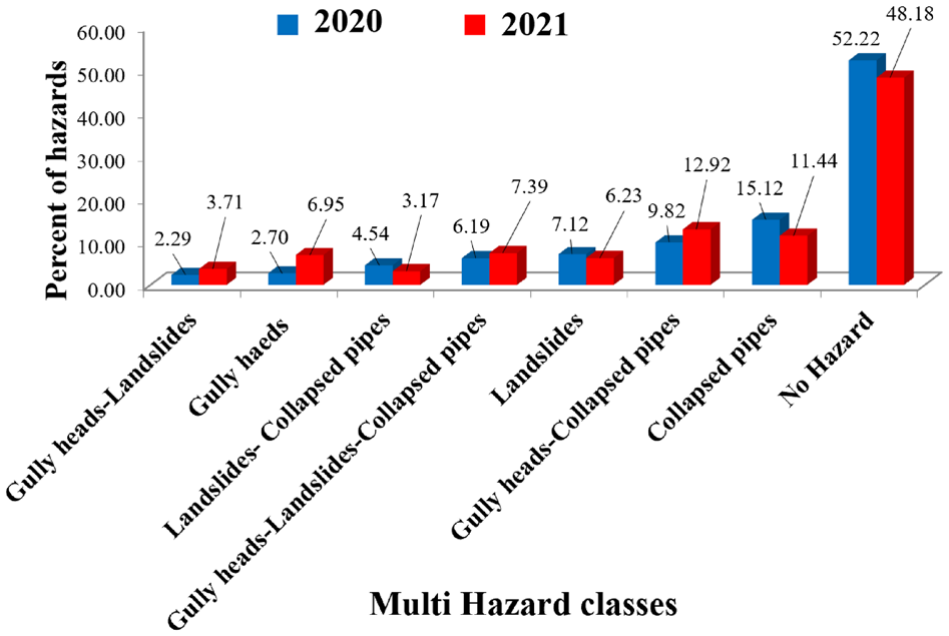
Percentage of each hazard in 2020 and 2021.

To prepare a multi-hazard map in 2020 and 2021, three higher accuracy susceptibility maps (one for each hazard) in 2020 and three higher accuracy susceptibility maps ((one for each hazard)) in 2021 were considered together (Fig. 3). The joint map in the form of a multi-event in 2020 and 2021 disclosed that most of the regions under study are not susceptible to any natural hazards, although a few percentages of regions are at hazardous risk of three natural events when analyzed jointly. Indeed, the results of the compound events analysis in 2020 and 2021 (Fig. 3) showed that 52.22% (in 2020) and 48.18% (in 2021) of the study area are not clearly susceptible to compound events, whereas 6.13% (in 2020) and 7.39% (in 2021) of the study area are susceptible to combined hazard of collapsed pipes, gully heads, and landslides (Fig. 4). The susceptibility classes of the compound events map in 2020 confirmed that collapsed pipes (15.12%) were the most frequent events in the study region, while collapsed pipes-gully heads (12.92) were the highest occurrences in 2021. Meanwhile, the percentage of the occurrence of three natural hazards (combined risk of studied events) in 2021 increased compared with 2020. However, the regions with no hazards decreased in 2021 compared with last year.

### Validation of natural hazard susceptibility maps

**Figure 5:**
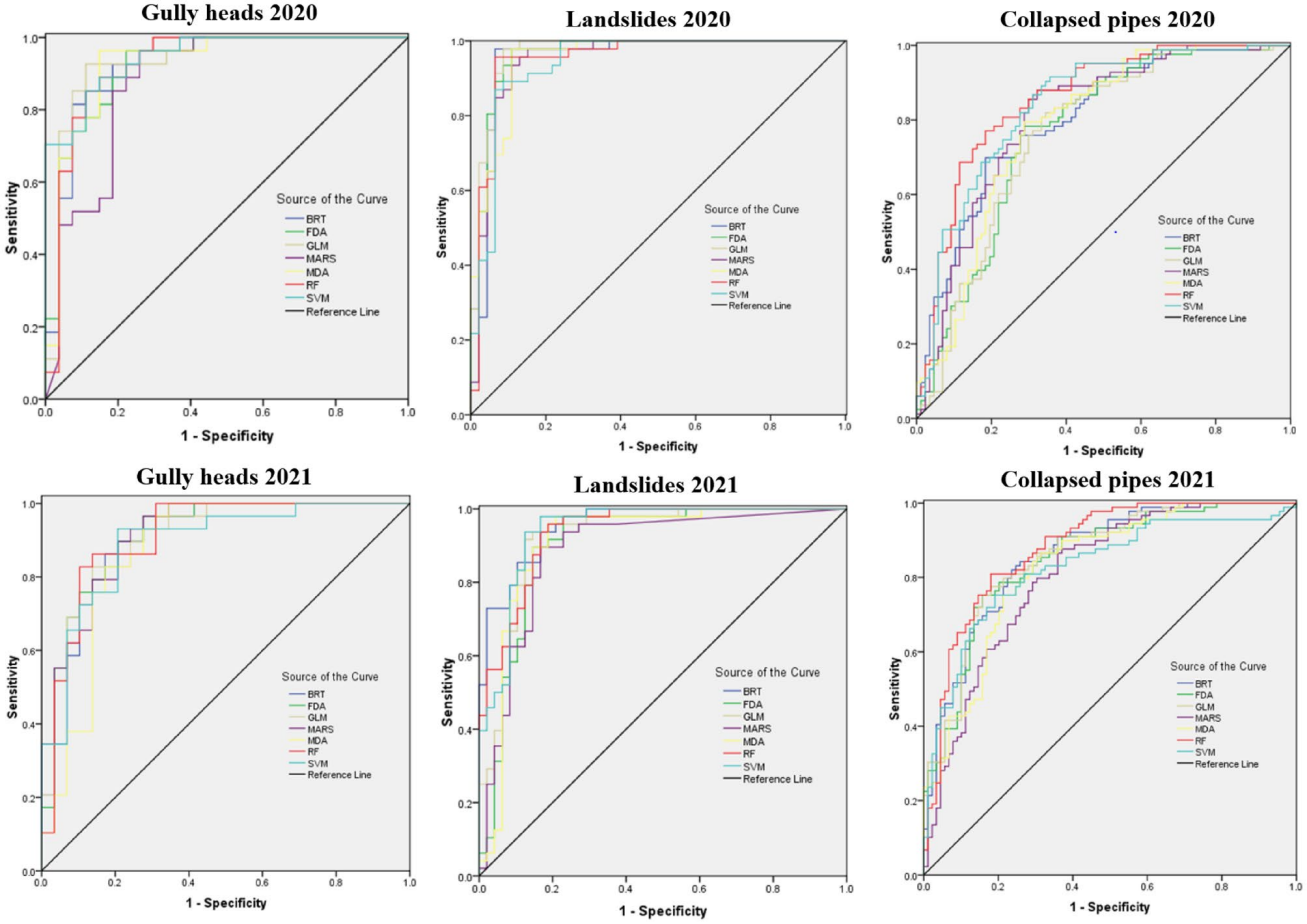
ROC curves for the seven models in the training step.

The accuracy of all maps provided by the BRT, FDA, MDA, SVM, RF, GLM, and MARS classifiers was verified by applying “ROC curves” (Fig. 5). The highest AUC values for each hazard map were different from one another. The data validation of the seven used models confirmed an excellent accuracy of the SVM, RF and GLM classifiers for gully heads, collapsed pipes, and landslides susceptibility maps in 2020, respectively. While, the MARS, RF, and BRT had the highest accuracy (excellent with more than 0.9%) for gully heads, collapsed pipes, and landslides susceptibility maps of 2021, respectively (Tables 1, 2, 3, 4, 5, 6, 7, 8 and 9). For instance, the AUC of SVM (0.948), GLM (0.969), and RF (0.855) in 2020 were the greatest for gully heads, landslides, and collapsed pipes, respectively (Tables 4, 5 and 6). Regarding 2021, MARS (0.914), BRT (0.955), and RF (0.881) had the highest value in the evaluation of gully heads, landslides, and collapsed pipes, respectively (Tables 7, 8, and 9). In the other words, the SVM and MARS models had excellent accuracy, especially regarding mapping gully heads, due to the strength of the model in data-driven aspects and non-linearity. Besides the low cost of the algorithm construction, it makes them exceedingly usable for predicting and assessing dynamic factors (i.e., anthropogenic or hydrological information). Other classifiers such as GLM and BRT, were more capable for prediction of landslides in 2020 and 2021, selected in this study because they consider nonlinear correlations among independent and dependent factors. The spatial accuracy of all models was more than 90% (excellent) for gully heads and landslides in 2020 and 2021, while it was very good (more than 80%) for collapsed pipes in the two studied years. Furthermore, all applied classifiers had excellent accuracy for predicting gully heads, collapsed pipes, and landslides in 2020 and 2021.

**Table 4:** Predictive performance of the seven applied models in the validation process of gully heads 2020.

Area under the curve					
Test result variable(s)	Area	Std. error^a^	Asymptotic sig.^b^	Asymptotic 95% confidence interval	
				Lower bound	Upper bound
BRT	0.926	0.038	0.000	0.852	1.000
FDA	0.923	0.037	0.000	0.851	0.996
GLM	0.934	0.037	0.000	0.849	1.000
MARS	0.879	0.049	0.000	0.782	0.975
MDA	0.927	0.038	0.000	0.850	1.000
RF	0.926	0.039	0.000	0.846	1.000
SVM	0.948	0.026	0.000	0.896	0.999

The test result variable(s): MARS has at least one tie between the positive actual state group and the negative actual state group. Statistics may be biased.

^a^Under the nonparametric assumption.

^b^Null hypothesis: true area = 0.5.

**Table 5:** Predictive performance of the seven applied models in the validation process of Landslides 2020.

Area under the curve					
Test result variable(s)	Area	Std. error^a^	Asymptotic sig.^b^	Asymptotic 95% confidence interval	
				Lower bound	Upper bound
BRT	0.952	0.026	0.000	0.895	1.000
FDA	0.964	0.020	0.000	0.922	1.000
GLM	0.969	0.018	0.000	0.922	1.000
MARS	0.949	0.025	0.000	0.900	0.998
MDA	0.953	0.021	0.000	0.911	0.994
RF	0.952	0.025	0.000	0.903	1.000
SVM	0.941	0.026	0.000	0.890	0.991

^a^Under the nonparametric assumption.

^b^Null hypothesis: true area = 0.5.

**Table 6:** Predictive performance of the seven applied models in the validation process of collapsed pipes 2020.

Area under the curve					
Test result variable(s)	Area	Std. error^a^	Asymptotic sig.^b^	Asymptotic 95% confidence interval	
				Lower bound	Upper bound
BRT	0.804	0.033	0.000	0.739	0.869
FDA	0.767	0.037	0.000	0.695	0.839
GLM	0.762	0.037	0.000	0.689	0.835
MARS	0.806	0.034	0.000	0.739	0.873
MDA	0.785	0.035	0.000	0.716	0.854
RF	0.855	0.029	0.000	0.798	0.912
SVM	0.839	0.031	0.000	0.778	0.899

^a^Under the nonparametric assumption.

^b^Null hypothesis: true area = 0.5.

**Table 7:** Predictive performance of the seven applied models in the validation process of gully heads 2021.

Area under the curve					
Test result variable(s)	Area	Std. error^a^	Asymptotic sig.^b^	Asymptotic 95% confidence interval	
				Lower bound	Upper bound
BRT	0.901	0.041	0.000	0.820	0.982
FDA	0.912	0.038	0.000	0.837	0.987
GLM	0.906	0.039	0.000	0.829	0.983
MARS	0.914	0.036	0.000	0.844	0.985
MDA	0.870	0.050	0.000	0.772	0.969
RF	0.910	0.040	0.000	0.831	0.989
SVM	0.892	0.043	0.000	0.808	0.976

^a^Under the nonparametric assumption.

^b^Null hypothesis: true area = 0.5.

**Table 8:** Predictive performance of the seven applied models in the validation process of landslides 2021.

Area under the curve					
Test result variable(s)	Area	Std. error^a^	Asymptotic sig.^b^	Asymptotic 95% confidence interval	
				Lower bound	Upper bound
BRT	0.955	0.018	0.000	0.919	0.990
FDA	0.903	0.034	0.000	0.836	0.970
GLM	0.922	0.029	0.000	0.865	0.979
MARS	0.886	0.037	0.000	0.814	0.958
MDA	0.910	0.035	0.000	0.841	0.978
RF	0.938	0.023	0.000	0.892	0.983
SVM	0.943	0.023	0.000	0.897	0.989

The test result variable(s): MARS has at least one tie between the positive actual state group and the negative actual state group. Statistics may be biased.

^a^Under the nonparametric assumption.

^b^Null hypothesis: true area = 0.5.

**Table 9:** Predictive performance of the seven applied models in the validation process of collapsed pipes 2021.

Area under the curve					
Test result variable(s)	Area	Std. error^a^	Asymptotic sig.^b^	Asymptotic 95% confidence interval	
				Lower bound	Upper bound
BRT	0.861	0.027	0.000	0.808	0.914
FDA	0.851	0.028	0.000	0.795	0.906
GLM	0.858	0.027	0.000	0.805	0.912
MARS	0.811	0.032	0.000	0.748	0.874
MDA	0.830	0.030	0.000	0.771	0.889
RF	0.881	0.025	0.000	0.831	0.930
SVM	0.827	0.032	0.000	0.766	0.889

^a^Under the nonparametric assumption.

^b^Null hypothesis: true area = 0.5.

## Discussion

### Multicollinearity analysis

This study investigated the corresponding factors of environmental variables prone to combinations of collapsed pipes, gully heads, and landslides using a linear regression model that allows comparing different events in one site. According to multicollinearity analysis, the first or second statistically standardized beta value among environmental covariables was recognized between collapsed pipes/gully heads and land use. Hosseinalizadeh et al.^11^ described that land use affects hydrological processes, which is erosional landform. These processes also affect subsurface water accumulation and make the material dissolve on the hardpan layer and eventually create sinkholes. Hydrological processes can also lead to the connection of a single sinkhole, which can lead to the formation of blind gullies that will eventually connect to the drainage network and form gully heads and gully network. In particular, an overland flow that is more than the substrate or soil infiltration capacity can be very adequate for initiating erosional processes (i.e., surface and inter-rill erosion) and slopes developing in regions with sparse land cover. It is exceedingly represented that the water and soil managers and landowners are forcefully linked. Moreover, the highest standardized beta value in analyzing gully heads/landslides was slope degree. Our fieldwork experiences showed that steeper slopes may increase a region's vulnerability to massive movements, including landslide forms. The steepness of slopes predominantly stimulates runoff velocity to increase through time, resulting in a vertical collapse in a gully channel bed or sudden displacement of sediments down the slope. Regarding slope, gully heads and landslides happen in the steep slopes of the central parts of the study area as stream density plays a significant role downstream of these slopes. The significant positive effects of slope and stream density on the occurrence of landslides have been reported by Hua et al.^44^.

### Multi-hazard maps produced using seven classifiers in 2020 and 2021

The main benefit of machine learning classifiers compared to other statistical-based methods is that they can easily solve the problem of noises in the dataset and are highly accurate in the confined measurement errors or the existence of uncertain data. However, seven classifiers and high accuracy imagery techniques were used to solve this problem. Well-documented advantages make the data mining classifiers, especially RF, which had the highest accuracy for predicting collapsed pipes in 2020, appropriate for monitoring the changes in combination with natural hazards. First, the applied classifiers are fast and straightforward, defined by great prediction performance^45^. For example, the RF algorithm provides an internally even-handed assessment of generalizability with a precise classifier in forest building and thus, prepares higher quality forecasts robustness^27^.

One of the best achievements of the present study regarding the studied combined hazards is the preparation of multi-hazard susceptibility maps in 2020 and 2021, which could very accurately predict the region susceptible to compound events, and thus, help us to focus our future researches here. Skilodimou et al.^46^ combined the statistical-based map of earthquakes, floods, and landslides and provide a single multi-hazard probability map. Yanar et al.^47^ provided a multi-hazard map of landslides and floods using the Mamdani fuzzy algorithm. Pourghasemi et al.^48^ studied the spatial behavior of compound events of flood, forest fire and landslide in Shiraz City, Iran and provided a multi-hazard map of interested hazards using the RF model. The multi-hazard susceptibility maps modeled in 2020 revealed that the places where all three hazards occurred together were less, but in 2021, this amount has increased. Thus, what is essential is the identification of factors or processes causing increasing these hazard occurrences at specific locations within the study area. Otherwise, a year should not impact increasing the occurrences of three combined hazards together.

Besides, collapsed pipes are the most dangerous hazard, followed by landslides and gully heads (see Fig. 4). Perhaps, the transformation of each of these hazards into one another leads to these results over one year. It means that the number of natural events that may occur cumulatively or simultaneously increases over time. The consequences of such event relationships occurring mean that an effect is generated which is a little bit different from that of the individual events occurring in isolation. This can lead to remarkable challenges, particularly for operators of national networks and/or asset managers. In other words, hazards are mostly behaved as independent or isolated. A multi-hazard alternative approach seeks to recognize all feasible natural events and their interrelationships. One natural event increase or triggers the possibility of one or more other events. For instance, a collapsed pipe may trigger gully heads, whereas a gully head may enhance the possibility of landslides being created soon.

Considering compound hazards and using the systematic collection of input maps, statistical classifiers, and high-resolution imagery tools can support adaptive management. From this perspective, multi-hazards assessment can increase our knowledge of Earth's internal and external processes, monitoring and forecasting of natural events and possible consequences when they occur coincidentally. They can clearly define the triggering hazardous landforms and their impacts on another due to various Earth surface and sub-surface processes that operated over short or long geological times. Further, finding how one event can increase or trigger the probability of a secondary event is essential to mitigating and investigating multi-hazard phenomena. It may force us to apply hazard interaction classifiers or matrices to realize links between natural events and describe potential cascading hazards based on scientific knowledge.

## Conclusion

Quantitative geomorphology is modeling and measuring the landform processes that shape the Earth's surface. Adopting the best controlling and managing plan for water and soil conservation will be possible whenever landforms and processes are also considered and recognized carefully. For example, collapsed pipes, gully heads, and landslides are responsible for considerable soil losses in arid and semi-arid regions and may not always be sufficient to monitor the effects of multi-hazards phenomena separately. In this study, the compound events susceptibility maps indicated damage resulting from compound events are enhanced in 2021 in comparison with the previous year, both in terms of occurrence and magnitude. Whereas the percentage of the region with no-hazards is decreasing as well. According to the AUC values, the best accuracy was specified for the GLM model for the prediction of landslide susceptibility map in 2020. Further, we attempt to examine multiple hazards instead of single approaches to achieve information on multi-hazard regions. Further, to be able to maintain and improve the sustainability of the environment and predict and reduce the effect of contemporary land surface and subsurface processes that lead to hazardous natural events (such as collapsed pipes, gully heads, and landslides), it is needed to answer the question of when and how big natural processes can be and where these compound events could occur. Besides, the effectiveness of the applied methods has been verified by the use of several statistical parameters and it resulted in quite good performances. Thus, the application of these methods is also suggestable for conservation purposes at national scales in another contexts, including in earthquake-and flood-prone areas.

## Methodology

**Figure 6:**
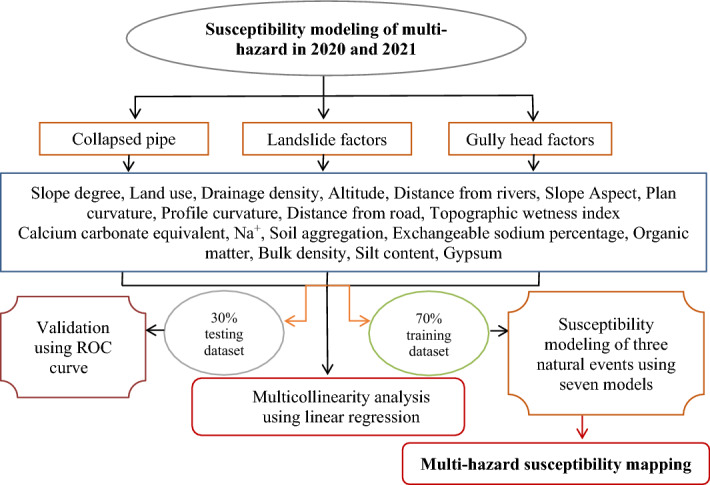
Methodology, approach and its components.

The data mining classifiers were used to analyze and to evaluate the spatial prediction of multi-hazards. The methodology, approach, and its components are shown in Fig. 6. The flowchart contains four main steps, including (1) data preparation in 2020 and 2021, i.e., obtaining the location of 281 collapsed pipes, 152 landslides, and 90 gully heads in 2020 and also 410 collapsed pipes, 328 landslides, and 198 gully heads in 2021 gathering in the field and unmanned aerial vehicles (UAV); (2) identification of the most hazardous environmental covariables come up with the occurrence of collapsed pipes, gully heads, and landslides using the linear regression algorithm; (3) spatial modeling of the collapsed pipes, gully heads, and landslides susceptibility along with validation processes applying seven data mining models; and, eventually, (4) preparing and comparing multi-hazards maps in 2020 and 2021. All statistical classifiers are exhaustively described in the researches cited below, hence, only a concise explanation is given here.

### Study area

**Figure 7:**
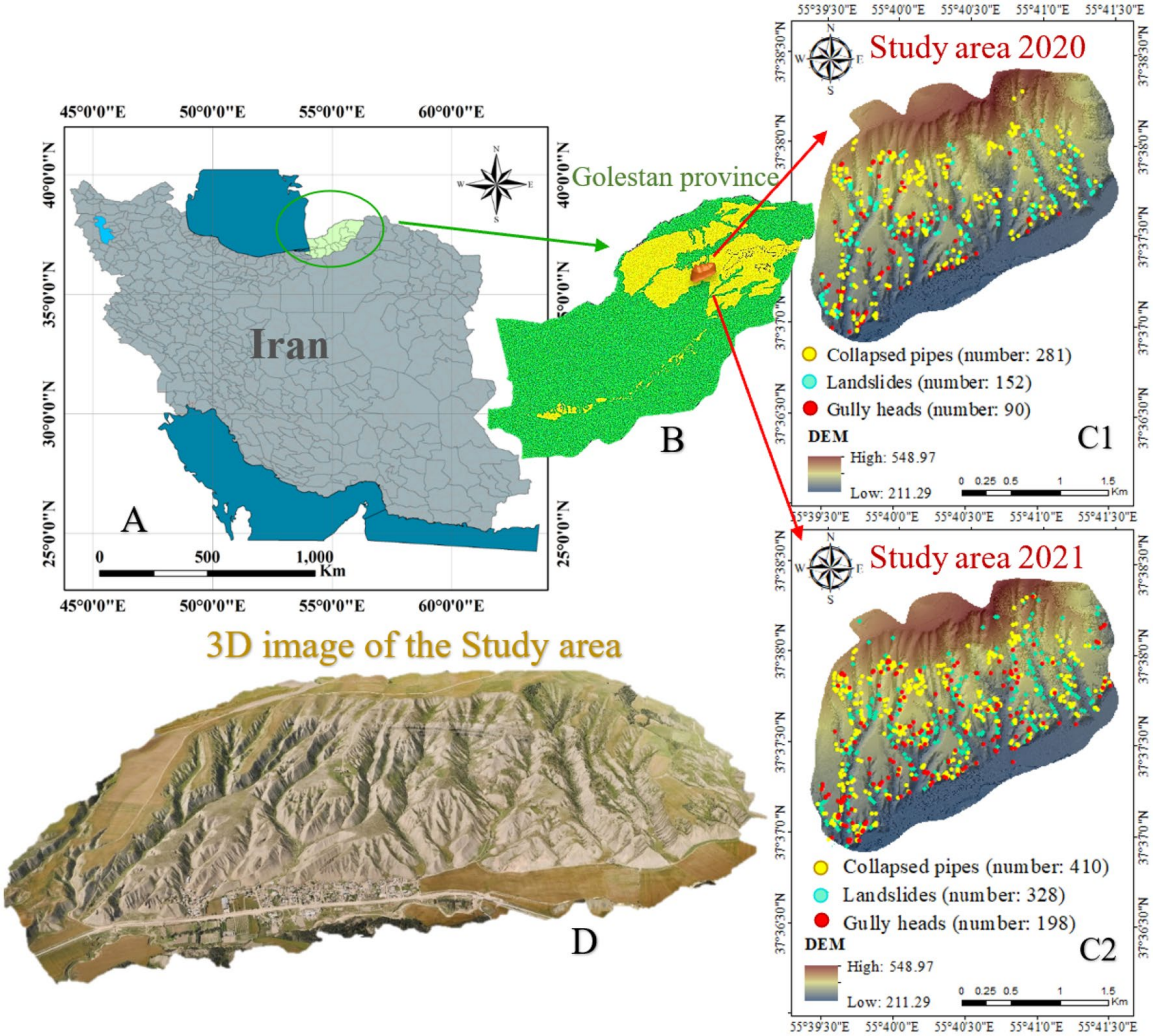
The location of study area in Iran (**A**), Golestan province (**B**), study area in 2020 (**C1**) and 2021 (**C2**), and the 3D map of the study area (**D**) prepared using ArcGIS 10.3.1 software (https://www.esri.com).

**Figure 8:**
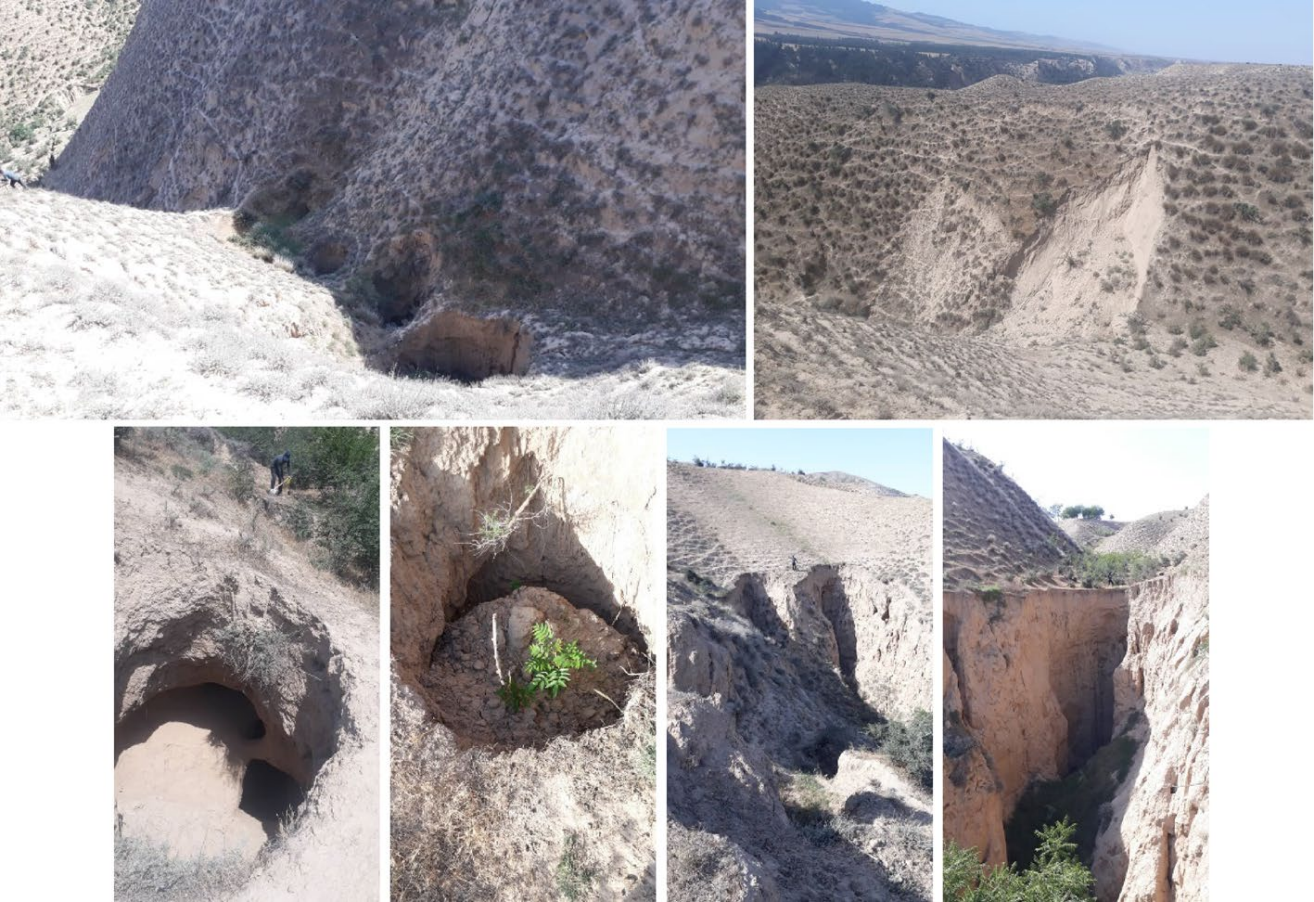
Some examples of three soil landforms (collapsed pipes, gully heads, and landslides) in the study area.

The study area is located in the “Golestan Province” (NE Iran) between 37″ 36′ 30° to 37″ 38′ 30° N latitudes and 55° 39′ 30° to 55° 41′ 30° E longitudes, with 520 ha of mainly arid and semi-arid regions. The study area was selected from the tributaries of Gorganrood with the loess-driven soils located in the east part of “Golestan province”. The maximum and minimum altitudes are approximately 548 and 208 m above sea level. The climate of semi-arid using the Domarten method, and the average annual rainfall is 260 mm reported by the Iran Meteorological Agency in 2021. The “silt loam” is the texture of the topsoil. The location map of the study region in Iran (a), Golestan province (b), and the study region (c) is shown in Fig. 7, and some examples of three landforms (collapsed pipes, gully heads, and landslides) showed in Fig. 8. Both overgrazing and intense farming contribute to developing the different types of soil erosion in this region. Besides, between the two calendar years, 2020 and 2021, intensive rainfall occurred for 7 days and nights in 03.17.2020 and ended on 03.24.2020. According to the “State Meteorological Agency”, the mean total rainfall was about 220 mm across the “Gorganrood watershed”, but four or five stations recorded up to 380 mm. That’s why, the numbers of each event increased, suddenly.

### Gathering data related to collapsed pipes, gully heads, and landslides hazards

The comprehensive mapping was done to recognize collapsed pipes, gully heads, and landslides. The spatial location of these three natural hazards was recorded applying UAVs (Sensefly eBee x) in 2020 and 2021. Unmanned aerial vehicles were applied with a camera model named Sensefly Aeria X and focal length of 18.5 mm. The average Ground Sampling Distance (GSD) was 5 cm. The orthomosaic resolution obtained was 5 cm/pixel. The trained UAV operator and the predefined flight paths were autonomously obtained by senseFly eMotion flight planning software. The flight paths were designed to have a front overlap of 85% and side overlap of 70%. The craft maintained a flight path 220 m above the surface. Aerial images acquired from the survey were processed with the Pix4Dmapper photogrammetry software. The gathered data were applied to re-check the exact positions of these natural events mapped during intensive fieldwork. The susceptibility mapping and monitoring tools used in 2020 and 2021 obtained the samples of non-hazards and three compound events to construct susceptibility maps and their evaluation. Of the total recorded compound events in the study area, 70% were applied in the basic phases of the model-building process, and the remaining were carried out in the validation phases in 2020 and 2021. Natural Breaks classification as an excellent method in ArcMap was used to classify the hazard/susceptibility maps^49^.

### Driving factors of collapsed pipes, gully heads, and landslides

Based on the literature review, the main covariables that trigger the occurrence of collapsed pipes and gully heads are broken up into four groups, including human factors (land use), topologic (altitude, slope degree), soil properties (calcium carbonate equivalent (CCE), organic carbon (OC), exchangeable sodium percentage (ESP), silt content, bulk density, soil stability), and hydrology (stream distance, stream density)^5,10,45–50^. Landslides are affected by a collection of anthropological (land use and road distance) and geo-environmental “(plan curvature, distance to streams, altitude, drainage density, profile curvature, topographic wetness index, and slope degree)” variables^44,45^. In other words, to assess the multicollinearity analysis between morphometric parameters and these three compound events, 9 (landslides), 11 (collapsed pipes), and 11 (gully heads) were chosen to add as independent variables. Topographical variables were extracted applying a “UAV-digital elevation model” (orthophoto images driven from the UAV) with a resolution of 1 m. Data layers were created applying ArcGIS 10.3.1 (https://www.esri.com). The distance to stream and distance to road maps were obtained from the roads and river maps. The land use map was also created from the orthophoto images. Finally, the number of 60 soil samples (the same sample size for each study landform and also for locations with no hazard; 15) was gathered in the field and transferred to the soil laboratory (detailed in Table 10).

**Table 10:** The physical–chemical soil properties of the location of collapsed pipes, gully heads, and landslides in the study area.

Soil properties	Minimum	Maximum	Average
Silt content	46	72	60.72
Organic carbon	0.7	1.7	1.10
ESP	0.66	2.68	1.05
Calcium carbonate equivalent	7.5	22	16.63
Soil stability	0.32	1.33	0.81
Bulk density	1.34	1.54	1.43

### Multicollinearity analysis

The linear regression algorithm was used to detect the multicollinearity of environmental covariables affecting three compound events. This fitting algorithm analyzes the interaction between the response variable (the absence or presence of gully heads, collapsed pipes or landslides) and independent variables^26–51^. The model's output represents the significantly (Sig) and coefficient calculated by SAS 16.0 software. By doing linear regression, “Variance Inflation Factor (VIF) and Tolerance (TOL)” were obtained to detect the multicollinearity among the corresponding factors and to define a noise which reduces the accuracy of the final model. Particularly, if TOL < 0.1 and VIF > 10, the predictor variable is multicollinear and should be omitted from the further modelling processes^52^.

### Support vector machine (SVM)

It divides different classes with an optimal hyperplane and thus, maximizes the spatial border between them. The points nearest to the hyperplane are named “support vectors” (the main components of the training dataset). These decision rules are performed by solving a quadratic optimization quandary solely. Further, the use of the classification concept separates classes and maximizes their spatial border^53^. This hyperplane with a higher spatial border has better generalization and is stable to noise. The SVMs, as a multi-layer perceptron^54^ remark a set of linear detachable training vectors and find an *n*-dimensional hyperplane and become different in the process of “two classes by their maximum gap”^55^. The function of the SVM response is described in Table 11.

**Table 11:** The function of models and their description.

Models	Function	Description
Support vector machine (SVM)	½ *W*^*2*^ Yi ((W_*_ Xi) + *b)* ≥ *1* ${\text{L }} = 1/2 \; W2 - \mathop \sum \limits_{i = 1}^{n} \lambda i \left( {Yi \left( {W* Xi} \right) + b} \right) - 1$	*W:* the normal of the hyperplane; *b:* scalar base; *λi:* the adjunction of Lagrange multipliers
Multivariate adaptive regression spline (MARS)	$max \left( {0, x - k} \right) or max \left( {0, k - x} \right)$ $y = f\left( x \right) = {\upalpha } + \mathop \sum \limits_{n = 1}^{n} B_{n} h_{n} \left( x \right)$	X: an independent variable; k: a constant corresponding to a knot; y: the dependent variable; βn and hn(x): an individual basis function

### Mixture discriminant analysis (MDA)

It combines more assembled neural network classifiers due to the “nonlinear nature of its classification rules”. Due to its modest structure, the MDA also makes straightforward interpretations in conjunction with linear mixture classifiers. The MDA proposes a new alternative for making three-dimensional models question or addressing ensemble modeling in remote sensing, typically from aircraft satellites^56,57^. As an advantage of MDA, we can add that it performs a mixture of classifiers with estimation using the Maximum Likelihood and expectation–maximization algorithm^58^.

### Flexible discriminant analysis (FDA)

It is a statistical analysis applying a discriminant function to assign data to one or more groups or to a non-parametric version differing in certain respects from an earlier one. It uses an optimal scoring method to post-processed a multi-response regression. In other words, the FDA procedure is one of the best classifiers for optimal data-scoring to illustrate the classes and canonically spatial correlation analysis. The FDA is equal to administering a linear discriminant analysis. Semi-parametric or non-parametric regression is replaced instead of the linear regression steps. Furthermore, the algorithm uses different regression tools and produces various class boundaries and distinction rules^59,60^.

### Random forest (RF)

It is a supervised classifier^61^ with relatively low error compared to previous classification classifiers and various decision trees. To split each node in this algorithm, the dummy codes use the minimum node dimension, and features^62^, although the predictor is the top splitter in all trees if one of the variables has more impact on the predicting function. Further, averaging predictions from correlated trees does not significantly reduce assumed variance, and all trees are constructed in a similarly correlated pattern^61^.

### Boosted regression tree (BRT)

It is an ensembled statistical-based algorithm that modifies an individual model by fitting and incorporating classifiers for the best prediction^33^. It is also combined boosting builds join with regression classifiers to reduce the amount of variance in a prediction and improve model accuracy. It makes the best or most effective use of the number of trees which set by inner bag fraction, cross-validation, and learning rate with no direct human control^34^. Eventually, the highest weakness of single tree classifiers (poor predictive implementation) may easily be solved by fitting multiple trees and rules of thumb^63^.

### Multivariate adaptive regression spline (MARS)

It is a statistical-based algorithm for fitting nonlinear complex communications between independent and dependent variables while providing an interpretable spatial model^38^. It works by splitting up the ranges of the expository parameters into areas by producing a linear regression function. The Breaks numbers between areas are named “knots”, although the term "basis function" implies each separate linear interval of the predictors. The basic equations as the response functions and the overall expression of MARS are described in Table 11^64^.

### Generalized linear model (GLM)

It linearly estimates the association between an event probability (collapsed pipes, landslides, or gully heads here) and predictors. It is also providing the possibility of an undemanding interpretation of coefficients. While considering a linear relationship between a predictor and its response is relatively unreal in some spatial modeling and may restrict predictive performance. The GLM can also assume nonlinear statistics by fitting nearly exact alteration functions to the predictors^42^. It has been illustrated as exceedingly beneficial in natural hazards, especially landslide modeling^41–65^.

### Evaluation of susceptibility maps

The “area under the receiver operating characteristic curve, or ROC curve”, illustrates the capability of classifiers (BRT, GLM, FDA, MDA, MARS, RF, and SVM in this study) to predict the susceptibility of the study area to collapsed pipes, gully heads, and landslides. The ROC curve indicates the tradeoff between the two rates. The values of this function reflect the accuracies in a range from poor to excellent^66^.

## Data Availability

The datasets generated and/or analyzed during the current study are not publicly available due [This work is ongoing in other parts] but are available from the corresponding author on reasonable request.
